# Scoping review of ethnobotanical studies on *Piliostigma thonningii (Schumach.) Milne-Redh.* in Sub-Saharan Africa

**DOI:** 10.3389/fphar.2025.1575548

**Published:** 2025-04-16

**Authors:** Bono Nethathe, Lonia Maanea Ramphinwa, Ananias Selekane Motadi, Frans Koketso Matlakala

**Affiliations:** ^1^ Department of Food Science and Technology, Faculty of Sciences, Engineering and Agriculture, University of Venda, Thohoyandou, South Africa; ^2^ Department of Plant and Soil Sciences, Faculty of Sciences, Engineering and Agriculture, University of Venda, Thohoyandou, South Africa; ^3^ Department of Nutrition, Faculty of Health Sciences, University of Venda, Thohoyandou, South Africa; ^4^ Research and Innovation Directorate, University of Venda, Thohoyandou, South Africa

**Keywords:** ethnobotanical medicine, malaria, *Piliostigma thonningii*, Sub-Saharan Africa, therapeutic uses, traditional uses

## Abstract

**Background:**

*Piliostigma thonningii* is a plant commonly used in traditional medicinal practices throughout Sub-Saharan Africa for the treatment of various ailments, such as respiratory infections, gastrointestinal and inflammatory disorders. Several studies have documented ethnobotanical uses of this plant in different countries in Sub-Saharan Africa.

**Aim:**

This study critically reviews the ethnobotanical uses mainly focusing on traditional medicinal uses of *P. thonningii* in Sub-Saharan Africa.

**Methods:**

A scoping review following the guidelines of Arksey and O’Malley was used to conduct this study. Various databases were used for searching for relevant articles and were handled in the reference manager EndNote. The data extraction focused on the links between *P. thonningii* and ethnobotanical uses in Sub-Saharan Africa. A descriptive analysis highlighted the years of publication, countries of publication, study designs, study participants, plant parts used, the diseases treated or managed, and how the plants are prepared or administered.

**Results:**

Of the 46 countries in Sub-Saharan Africa, only 14 have published studies on the ethnobotanical uses of *P. thonningii*. Notably, Nigeria emerged as a leader in this field, with the most publications. The results also highlight that leaves are the most common part of the plant used and that remedies are mostly prepared as a decoction, with the plant being popularly used to treat malaria.

**Conclusion and recommendation:**

This scoping review provides a comprehensive overview of traditional healing practices using *P. thonningii* in Sub-Saharan Africa and reveals substantial knowledge gaps across the region. However, the study revealed limitations such as lack of standardization on methods used to prepare remedies and dosages.

## Graphical Abstract

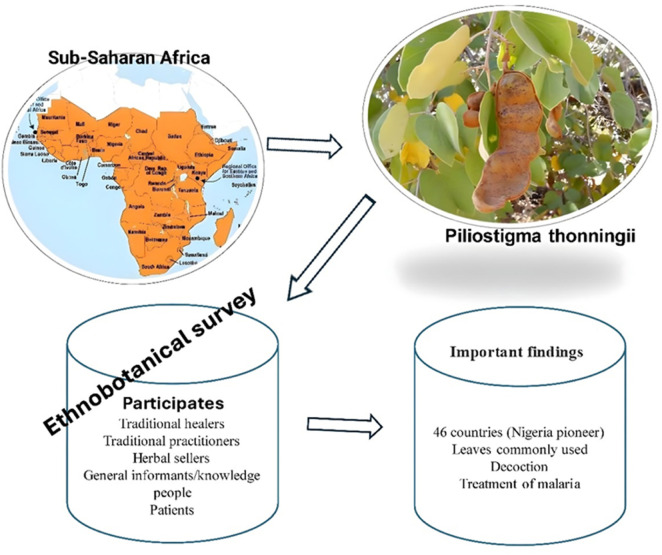

## Introduction

**FIGURE 1:**
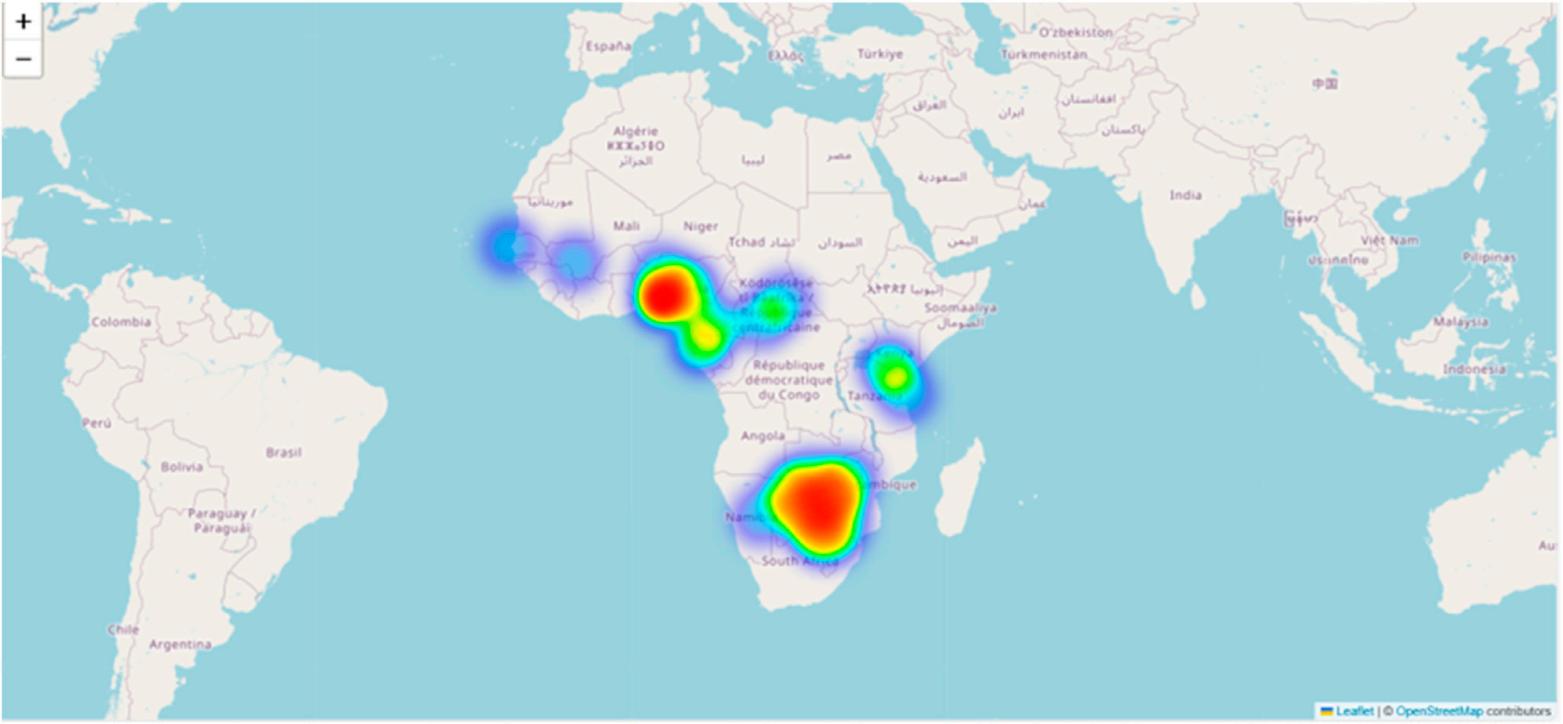
A heat map showing areas where *Piliostigma thonningii* is found.

*Piliostigma thonningii (Schumach.) Milne-Redh.* belongs to the family Fabaceae and sub-family Caesalpinioideae and is a native of tropical Africa (Moraa, 2023). It is widely distributed in Botswana, Ethiopia, Kenya, Sierra Leone, South Africa, and Swaziland (Figure 1). In South Africa, it is restricted to the northeastern part of Mpumalanga, but it is more abundant in open woodland vegetation in Limpopo Province. Common/local names in Africa include wild bauhinia, Rhodesian bauhinia, camel’s foot (English), mchekeche, mchikichi (Swahili), ihabahaba (Ndebele), mutukutu (Shona), mukura (Embu) and kigali (Luganda) (Orwa et al., 2009). While in South Africa, its common names are kameelspoor (Afrikaans.); mukolokote (Venda); mokgoropo (North Sotho) and nkolokotso (Tsonga) (Mogale et al., 2019).

**FIGURE 2:**
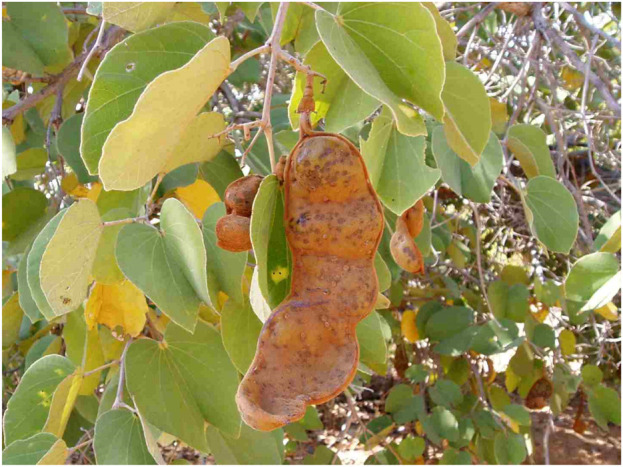
*Pilliostigma thonningii* tree with large, thick, reddish brown, non-splitting pods, picture obtained from (Bingham et al., 2025).

*Piliostigma thonningii* is a deciduous or evergreen tree or shrub with a rounded crown and a single stem (Tittikpina et al., 2016). It can grow 3–15 m tall and has a short bole which is often crooked (Moraa, 2023). It is a multipurpose tree collected from the wild that provides medicine, food, firewood, and a range of commodities for the local people (Moraa, 2023). The flowers have five white to pink petals and bloom from December to February. The female and the male flowers commonly occur on different trees. However, if the flowers are on the same tree, male flowers bloom first and then female flowers later to avoid so self-pollination (Orwa et al., 2009). Flowers are followed by thick, large, reddish brown, and non-splitting pods about 30–70 mm long (Figure 2) (Moraa, 2023; Orwa et al., 2009). Its bark has a rough surface and dark brownish-grey in colour. A visible feature of the tree is its simple, large, two-lobed, leathery leaves, which bear a resemblance to a camel’s foot, hence its common name (Figure 2) (Moraa, 2023; Orwa et al., 2009). The fruit is hard, hairy, and the pulp of *P. thonningii* contains a number of nutritional components that can be extracted, including vitamins, proteins, calcium oxalates, organic acids, fiber, starch, and essential oils (Orwa et al., 2009).

*P. thonningii* is part of traditional medicinal plants that have been known to treat a broad array of ailments globally. For instance, in Turkey, other plant taxa such as *Tilia rubra* subsp. *caucasica*, *Prunus laurocerasus*, *Urtica dioica, Pinussylvestris* var. *hamata*, *Thymus praecox* subsp. *Grossheimii, Galium palustre, Pilosella leucopsilon subsp. pilisquama, and Astragalus nitens and* Plantago *lanceolata* are used to treat respiratory tract disorders, skin diseases, digestive disorders, and other diseases (Bingham et al., 2025; Karaköse, 2022). Furthermore, traditional medicinal plants also play a vital role in the treatment of different diseases in livestock (Şen et al., 2022).

*P. thonningii* constituents are responsible for the treatment of ulcers, diarrhoea, heart conditions, and gastric pains, among other ailments, by traditional medical practitioners in Africa. This plant`s leaves, bark, roots, and pods have played an important role in traditional medicine. The knowledge of traditional uses of the plant has been passed down through generations (Güler et al., 2021; Mahomoodally, 2013). In recent years, there has been an increase in research to assess the nutraceutical and pharmacological potentials of the plant. This interest is prompted by a larger trend to investigate traditional medicinal compounds for possible use in Western healthcare (Afolayan et al., 2018). Some of the active compounds known to be employed in the treatment of different ailments are anthraquinones, flavonoids, tannins, saponins, steroids, 22 volatile oils, alkaloids, and terpenoids (Abubakar et al., 2024).

However, there is a lack of scoping reviews on *P. thonningi*`s ethnobotanical uses, especially in the African context. Therefore, as a first stop, the aim of this scoping review is to document the ethnobotanical use of *P. thonningii* for healthcare in Sub-Saharan Africa. Sub-Saharan Africa is known for its diverse languages, ethnicities, cultures, and a rich tapestry of indigenous healing practices (Cyril et al., 2021). The review systematically outlines the various modalities employed in traditional medicine and discusses the implications of these findings for public health in Sub-Saharan Africa.

## Methods

### Research approach

This scoping review followed the guidelines of Arksey and O’Malley (Arksey and O’malley et al., 2024) on the steps of conducting scoping reviews. The scoping review was used due to its ability to explore broader topics by mapping the existing literature (Arksey and O’malley et al., 2024) and making recommendations for future studies. The first step was to identify the research question to ensure it aligned with the study aim. In developing the research question, we adopted the PEO (population, exposure, outcome) framework in order to ensure that it was topic-focused and aligned with the objective of the scoping review (Arksey and O’malley, 2005) (Table 1). The research question was phrased as: What are the documented ethnobotanical uses of *P. thonningii* in Sub-Saharan Africa?

**TABLE 1:** Frameworks applied to the development of your research question.

Objective 1	Framework	Description
To identify and document the traditional uses of *Piliostigma thonningii* across different cultures and regions	PEO	P= *Piliostigma thonningii* E = Traditional uses of the plant O = Documented medical uses of the plant

A comprehensive search strategy was employed to identify relevant studies across multiple databases. The review used databases such as Web of Science, EBSCOhost, Science Direct, Springer Nature, and Google Scholar. The search utilized a combination of keywords and Boolean operators, truncations, and MeSH terms to widen the search. The developed search string was: (pilliostigma thonningii OR “camel foot tree” OR “Piliostigma thonningii” OR Schumach) AND (Milne-Redh) AND (traditional uses OR ethnobotanical medicines OR indigenous uses OR therapeutic uses) AND (Sub-Saharan Africa). This search string was used in the identified databases, and to align to each database’s advanced search, we requested the assistance of a faculty librarian as it is recommended for review studies (Tricco et al., 2024). Before exporting articles to a reference manager, EndNote 21, we developed inclusion and exclusion criteria. We planned to include peer-reviewed journal articles that covered *P. thonningii* and ethnobotanical survey on Sub-Saharan Africa. Studies published in the english language, addressed preventive and curative aspects of *P. thonningii*, and explored traditional/indigenous uses looking at healthcare. Furthermore, the study design of the included studies was published between 2020 and 2024.

We excluded studies that focused on animals and were conducted outside of Sub-Saharan Africa. In addition, unpublished dissertations and thesis, commentaries on studies, letters to the editor, systematic, scoping, and narrative reviews, and studies whose full lengths could not be accessed were excluded. Also, studies with insufficient results suitable for analysis were excluded.

**FIGURE 3:**
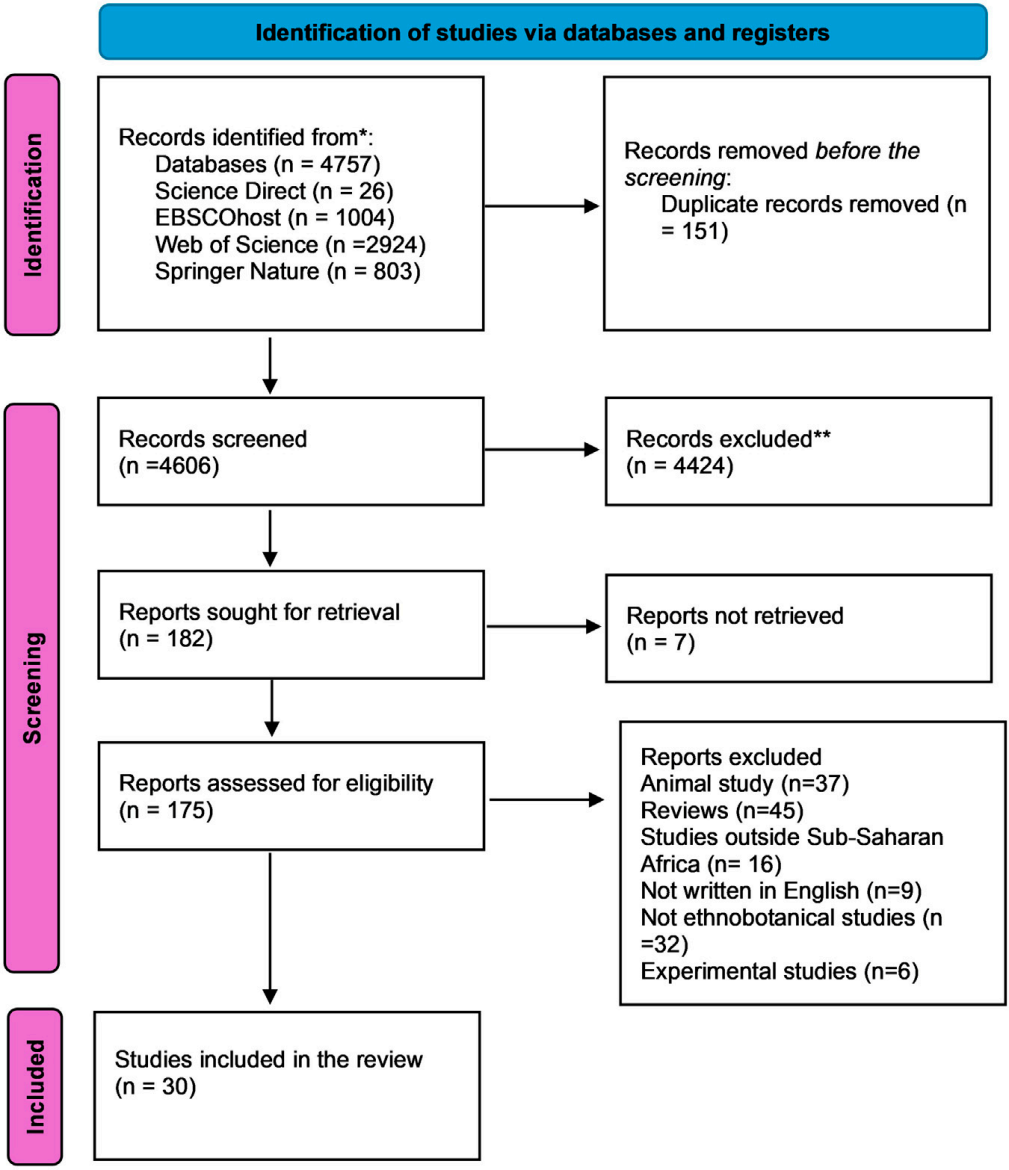
PRISMA-ScR diagram showing how the screening and selection process was conducted.

Articles that met the inclusion criteria were exported to the reference manager in preparation for performing screening. The first step was to combine all articles from different databases in the reference manager so that duplicates could be removed. After removing the duplicates, three reviewers (BN, MR, and SAM) divided amongst themselves articles to perform title and abstract (T&A) screening with one reviewer (FK) serving as the mediator and monitor of the process. After performing T & A screening on reference manager, articles that met the inclusion criteria were moved to another group on EndNote titled full-text screening (see Figure 3 for the summarised process). We then applied the option “retrieve pdfs” in the group “full-text screening.” Articles that could not be retrieved from initial databases were subsequently accessed via Google Scholar. After gathering all the articles, a data chart was created in Microsoft Word to facilitate data synthesis. Articles not meeting inclusion criteria during full-text screening were removed from the reference manager group tab, and those meeting the inclusion criteria were added to the data charting (Supplementary Table S1).

After charting the data, the data was analysed using descriptive analysis. A descriptive analysis was also conducted highlighting the years of publication, countries of publication, vernacular names, study participants, plant parts used for making remedies, disease treatment/management, and mode of preparation and administration. The findings were presented in the table (Table 2). Moreover, before producing this article, we used PRISMA-ScR checklist study (Arksey and O’malley, 2005) for rigor in the study.

**TABLE 2:** Ethnobotanical and ethnopharmacological applications of *Camel's foot tree (Piliostigma thonningii)* in Sub-Sahara Africa.

Study no	Author (year of publication)	Country	Vernacular name	Participates	Part of the plant used	Diseases treated/managed	Mode of preparation and administration in which the plants are used
1.	Hailemariam et al. (2021)	Ethiopia	Abafe	72 general local informants and 12 key informants	Bark	Liver diseases	Fumigation
2.	Toafode et al. (2022)	Republic of Benin	—	Traditional healers who specialize in the treatment of bone fractures and associated complications (n = 60)	Leaves, roots	malaria, leprosy, wounds, ulcers, gingivitis, fever, cough, toothache, sore throat, dysentery, diarrhoea, inflammation, skin diseases, and intestinal problems	Infusion, powdered
3.	Akwongo et al. (2022)	Uganda	Ogali	Herbalists and knowledgeable people n = 63	Roots, barks	Oropharyngeal candidiasis	maceration/decoc- tion: can remove the epidermal layer, pound, mix with little water/ boil & cool, sieve Admn: orally: 1 4 or 2 tsp/ 5/10/15 mls once/twice/ thrice a day. Can mix little filtrate with food; anally: 3 ml once a day
4.	Chegaing et al. (2020)	Cameroon	—	Traditional healers (n = 41)	Leaves, stem bark	Rheumatism, fever, gastritis	Decoction
5.	Evbuomwan et al. (2023)	Nigeria	Abafe	Registered traditional medicine practitioners n = 35	Stem bark	Malaria	—
6.	Ouédraogo et al. (2020)	Burkina Faso	Nabali (Gourmantché), Bag gnanga (Mooré)	Herbal traders selling medical plants in the markets n = 30	Leaves	Diarrhea, dysentery	Drink, bath
7.	Masumbu et al. (2023)	Malawi	—	25 Traditional herbal practitioners	Root, bark	Different types of cancer (prostate, tumor, chronic ulcers)	Decoction, powder Oral, topical
8.	Dery et al. (2023)	Ghana	Hanbome	50 informants	Leaves	Snake Bite, Wounds Stomachache, Sore throat, Hemorrhage.	topical, infunsions, oral/inhalation and Decoction
9.	Kola et al. (2020)	Togo	Eclo/Ew´e; Babakou/Kaby	Traditional healers n = 50	Leaves, roots	Skin, lung, chronic wound, prostate cancer	Decoction, sauce, powder Oral, topical
10.	Dossou et al. (2022)	Benin	—	Traditional healers n = 238; Major knowledge holders n = 56	Leaves	Snakebite	sacred rings/ amulets worn to avoid being bitten or approached by a snake
11.	Valentin et al. (2020)	Democratic Republic of Congo	Tshifumbe, Tshiluba, kifumbe	Informant`s practitioners of traditional medicine n = 84	Leaves	Malaria	Decoction Drink half a glass 2x a day for 4 days
12.	Valentin et al. (2022)	Democratic Republic of Congo	Kifumbe	Traditional medicinal healers n = 50	Stem bark	Typhoid fever, gastritis, haemorrhoid, cough.	Maceration for 72 hours of 5 handfuls of stem bark in 1 L of water. Drink 100 mL three times a day for 3 days
13.	Valentin et al. (2023)	Democratic Republic of Congo	Tshifumbe	Herbalists n = 80	Stem bark	Diabetes Mellitus	Decoction
14.	Jeane et al. (2020)	Côte d'Ivoire	Koukan	Traditional helears n = 15	Fruit	Malaria	Oral, bath
15.	Bamogo et al. (2023)	Burkina Faso	Bagendé/Niama yiiri	Traditional health practioners n = 17	Flowers/Raw	Snake Repellent	Fresh/Swallow a bulb for 12 months of protection
16.	Herve et al. (2023)	Cameroon	Kekame (Tikar)	Traditional healers n = 9	Barks	Chronic wounds	Spraying
17.	Valetin et al. (2024)	Democratic Republic of Congo	Kifumbe (Bemba)	households (*n* = 2730), herbalists (*n* = 48), traditional practitioners (*n* = 128)	Leaves, root bark	Wound, arthritis, malaria, diarrhea, gingivitis	Powder
18.	Namukobe et al. (2021)	Uganda	Kilama (Lus)	63 respondents	Leaves	Chickenpox	Powder mixed with jelly applied
19.	Ndodza et al. (2020)	Nigeria	Obleigbo	Informants n = 79	Leaves	Snakebites	Oral
20.	Dossou et al. (2021)	Benin	Kparounmon (Idaatcha,Nagot), Baroupkapka (Bariba), Kloman (Fon,Mahi), Aklo (Adja) Barkehi (Peulh)	339 people Traditional healers n = 285 Hunters n = 54	Roots, Leaves, Bark	Snakebites	Decoction, Pounding, Incineration
21.	Sidiq et al. (2022)	Nigeria	Abafe	53 respondents Speciality n = 16 Traditional medical practitioner = 14 Herb seller = 23	leaves	stroke	—
22.	Kankara et al. (2022)	Nigeria	Kalgo	Respondents comprising traditional healers, herbalists, farmers and HIV/AIDS patients n = 150	Bark	Bowel Infection	Powder/oral
23.	Kroa et al. (2022)	Ivory Coast	Gnanman (Dioula); Tchanhanm (Senoufo)	251 people, including practitioners of traditional medicine n = 51 and people from the general population n = 200	Sheets; fruit; roots	Malaria	Decoction; Oral route; bath
24.	Diop et al. (2022)	Côte d’Ivoire	kéoukégba	290 knowledge holders	—	Malaria	—
25.	Danjuma et al. (2022)	Nigeria	Kalgo	A total of 46 TMPs Traditional medicine practitioners	Stem bark	Jaundice	Dried and ground to powder. The powder is mixed with pap and consumed twice daily for 1 week
26.	Traore et al. (2022)	Guinea	Barkè (Pular) Yorokoye (Sousou)	three hundred and forty-nine Traditional Health Practitioners (THPs) respondents, including 244 traditional healers and 105 herbalists	Leaves, root bark	Hypertension	Decoction
27.	Lawal et al. (2022)	Nigeria	—	100 participants	—	Potency	—
28.	Bolou et al. (2022)	Côte d'Ivoire	gnamanbou (malinké)	herbalists in nine markets	Leaves	Chicken pox	Decoction beverage, bath and enema two times a day
29.	Falana et al. (2023)	Nigeria	Abafe	—	—	Skin conditions	—
30.	Mohlakoana and Moteetee (2021)	South Africa	—	—	Stem bark	Dysentery, wounds, respiratory ailments, snake bites, hookworms and skin diseases, chronic ulcers, diarrhoea, toothache, gingivitis, cough and bronchitis, used as soap.	—

The above figure (Figure 3) shows that the search identified 4,757 records, which were downloaded into Endnote. After removing duplicates, 4,606 records were left. After screening titles and abstracts, 175 articles were eligible for full-text screening. Thereafter, screening of the full articles was conducted; 145 articles were excluded either because full articles were not available, the focus of the study was not Sub-Saharan Africa, or the study was not related to human health, leaving 30 articles.

## Results

### Contextualisation of studies

**FIGURE 4:**
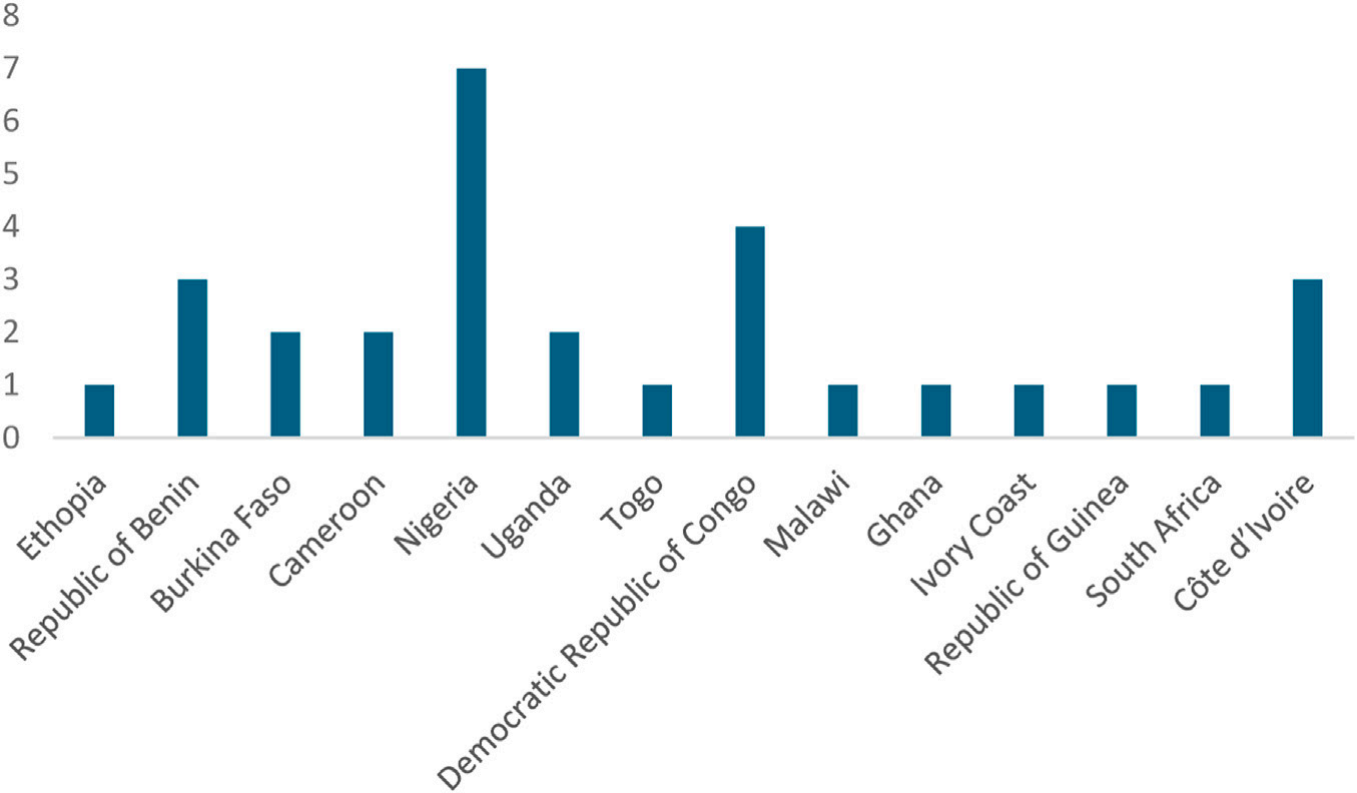
Distribution of ethnobotanical studies in Sub-Saharan Africa.

The search resulted in the inclusion of 30 articles. Sub-Saharan Africa has 46 countries, and only 14 countries have publications on ethnobotanical studies of P. thonningii. These countries include Ethiopia (Hailemariam et al., 2021), the Republic of Benin (Dossou et al., 2021; Dossou et al., 2022; Toafode et al., 2022), Burkina Faso (Ouédraogo et al., 2020; Dossou et al., 2021), Cameroon (Chegaing et al., 2020; Herve et al., 2023), Nigeria (Sidiq et al., 2022; Ndodza et al., 2020; Danjuma et al., 2022; Lawal et al., 2022; Kankara et al., 2022; Evbuomwan et al., 2023; Falana et al., 2023), Uganda (Byamukama et al., 2021; Akwongo et al., 2022), Togo (Kola et al., 2020), the Democratic Republic of Congo (Bashige et al., 2020; Bashige et al., 2022; Bashige et al., 2023; Bashige et al., 2024), Malawi (Masumbua et al., 2023), Ghana (Dery et al., 2023), Ivory Coast (Kroa et al., 2022), Côte d'Ivoire (Bolou et al., 2022; Diop et al., 2022; Jeane et al., 2020), Guinea (Traore et al., 2022) and South Africa (Mohlakoana et al., 2021). The country with the most publications was Nigeria, with 7, and the countries with the least publications were Ethiopia, Togo, Malawi, Ghana, Ivory Coast, Guinea, and South Africa, with only 1 publication (Figure 4; Table 2).

### Study designs used

The studies encompassed only one study design, which was ethnobotanical studies, which were studies focusing on the *P. thonningii*, traditional practices, and medical uses of plants by local communities. Ethnopharmacological studies were also included, investigating traditional knowledge related to the medicinal uses of *P. thonningii* within specific cultural or ethnic groups in Sub-Saharan Africa (Chegaing et al., 2020; Jeanne et al., 2020; Sidiq et al., 2022; Nodza et al., 2020; Ouédraogo et al., 2020; Kola et al., 2020; Byamukama et al., 2020; Mohlakoana et al., 2020; Dossou et al., 2021; Hailemariam et al., 2021; Bashige et al., 2021; Akwongo et al., 2022; Bolou et al., 2022; Danjuma et al., 2022; Diop et al., 2022; Dossou et al., 2022; Lawal et al., 2022; Toafode et al., 2022; Traore et al., 2022; Kankara et al., 2022; Kroa et al., 2022; Bashige et al., 2020; Dery et al., 2023; Evbuomwan et al., 2023; Falana et al., 2023; Herve et al., 2023; Masumbu et al., 2023; Bashige et al., 2023; Bashige et al., 2024) (Supplementary Table S1).

**FIGURE 5:**
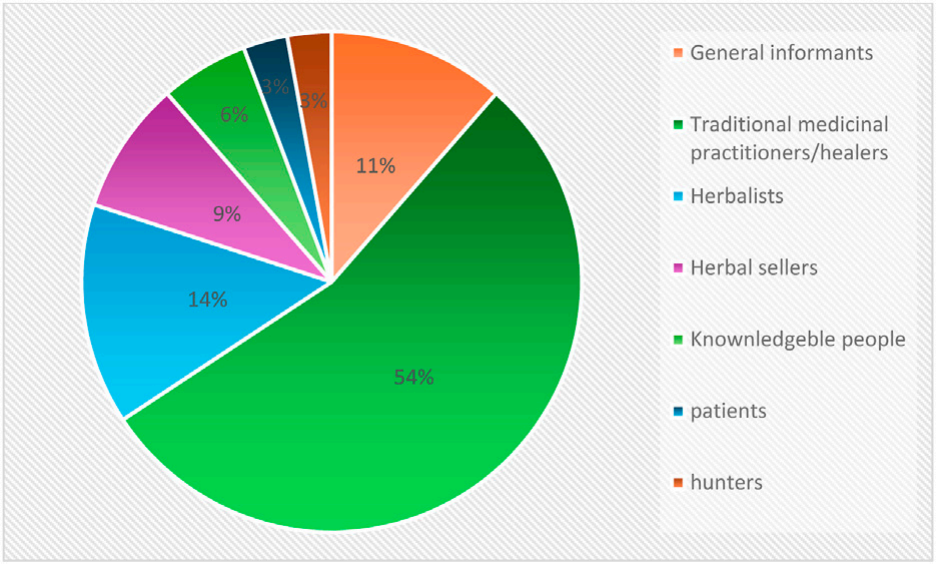
Distribution of ethnobotanical survey participants.

Furthermore, the participants used in ethnobotanical survey across sub-Saharan Africa differ slightly. The largest group is traditional medicinal practitioners/healers (n = 54%) (Chegaing et al., 2020; Jeanne et al., 2020; Kola et al., 2020; Dossou et al., 2021; Akwongo et al., 2022; Danjuma et al., 2022; Dossou et al., 2022; Toafode et al., 2022; Traore et al., 2022; Kankara et al., 2022; Kroa et al., 2022; Valentin et al., 2022; Sidiq et al., 2022; Bamogo et al., 2023; Evbuomwan et al., 2023; Herve et al., 2023; Masumbua et al., 2023; Valentin et al., 2023; Valentin et al., 2024), general informants (n = 11%) (Nodza et al., 2020; Valentin et al., 2020; Byamukama et al., 2021; Hailemariam et al., 2021; Kroa et al., 2022; Lawal et al., 2022; Dery et al., 2023; Valentin et al., 2024) herbal sellers (n = 9%) (Ouédraogo et al., 2020; Sidiq et al., 2022) knowledgeable people n = 6%) (Akwongo et al., 2022; Dossou et al., 2021; Diop et al., 2022) and patients (n = 3%) (Kankara et al., 2022) (Figure 5).

### Frequency index of parts of *P. thonningi* used for medicinal purposes

**FIGURE 6:**
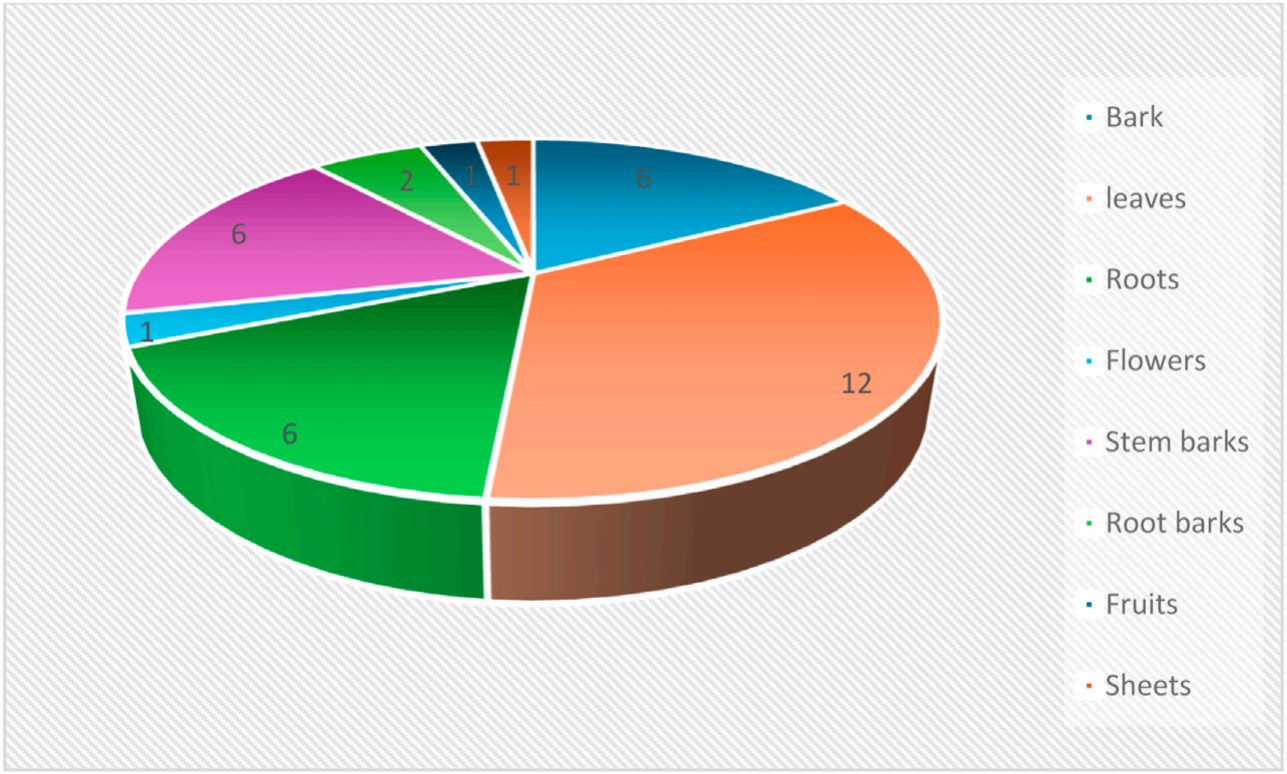
Frequency index of parts of *P.thonningii* used for medicinal purposes.

The parts of *P. thonningii* used for medicinal purposes were leaves, barks, roots, stem bark, root bark, flowers, fruits, and sheets. Figure 6 shows that leaves were the most used part, followed by bark, stem bark, and roots and the least used were sheets, fruits, and flowers.

Different plant parts were prepared in different ways and used as remedies. They were prepared as a decoction, powder, juice, macerated or taken with food, taken as raw fruit. Table 2 shows frequency index of how the remedies were prepared, fumigation (n = 1) (Hailemariam et al., 2021), infusion (n = 2) (Toafode et al., 2022; Dery et al., 2023), decoction (n = 11) (Chegaing et al., 2020; Kola et al., 2020; Valentin et al., 2020; Dossou et al., 2021; Dery et al., 2023; Akwongo et al., 2022; Bolou et al., 2022; Kroa et al., 2022; Traore et al., 2022; Valentin et al., 2023; Masumbua et al., 2023), powder (n = 7) [Kola et al., 2020; Byamukana et al., 2021; Danjuma et al. (2022)] maceration (n = 2) (Akwongo et al., 2022; Valentin et al., 2022; Kankara et al., 2022; Toafode et al., 2022; Masumbu et al., 2023; Valentin et al., 2024) and taken as raw fruits (n = 1) (Bamogo et al., 2023). The remedies were used for bathing, as an enema, for infusion, as a fumigant, or for gargling, spraying, drinking, swallowing, worn as scared rings/amulets, and were applied topically.

### Diseases treated by *P. thonningii* in sub Saharan Africa

The remedies were used to manage general symptoms such as liver diseases (Hailemariam et al., 2021), malaria, leprosy, wounds, ulcers, gingivitis, fever, cough, toothache, sore throat, dysentery, diarrhoea, inflammation, skin diseases, and intestinal problems (Chegaing et al., 2020; Ouédraogo et al., 2020; Valentiin et al., 2020; Toafode et al., 2022; Dery et al., 2023; Evbuomwan et al., 2023) oropharyngeal candidiasis (Akwongo et al., 2022), rheumatism and gastritis (Chegaing et al., 2020; Valentin et al., 2022), cancer (Kola et al., 2020; Masumbu et al., 2023), snake bites stomach-ache and hemorrhage (Nodza et al., 2020; Dossou et al., 2021; Dossou et al., 2022; Dery et al., 2023), typhoid fever (Bolou et al., 2022), diabetes mellitus (Valentin et al., 2022), snake repellent (Bamogo et al., 2023), arthritis (Valentin et al., 2024), chicken pox (Byamukana et al., 2021; Bolou et al., 2022), stroke (Sidiq et al., 2022), bowel infection (Kankara et al., 2022), jaundice (Danjuma et al., 2022), hypertension (Traore et al., 2022), potency (Lawal et al., 2022), respiratory ailments, hookworms, bronchitis (Mohlakoana and Moteetee, 2021). The results revealed that the 3 most common ailments treated by this plant were malaria (n = 7), snakebites (n = 5), and wounds (n = 5) (Table 2).

## Discussions

The significant strengths of this scoping review lie in its vigorous assessment of research on ethnobotanical and ethnopharmacological surveys of *P.thonningii* medicinal purposes in humans focusing on sub-Saharan Africa. Sub-Saharan Africa is known for its reliance on traditional medicinal practices. This practice has been passed down from generation to generation (Valentin et al., 2024). The results of the study showed that over time, the use of *P. thonningii* has increased, and people rely on it to treat various ailments, hence an increase in research and extensive exploration of P. *thonningii*. However, the study had some limitations such as the studies included were only the publications that had full text available and accessible, written in English, and the time ranged from 2020 to September 2024, employed a search limit of five databases. These may have inadvertently excluded some research publications on *P.thonningii* used in sub-Saharan Africa since researchers in the sub-region also publish in French and other languages. Despite these limitations, the present study provides valuable insights into the subject matter.

In order to document traditional knowledge about the plant’s therapeutic use within particular cultural contexts, ethnobotanical and ethnopharmacological methodologies mostly relied on interviews. However, the lack of standardized procedures, particularly in dosage and preparation, is one of these studies’ weaknesses because it makes comparisons and generalizations more difficult. Therefore, more research is required to overcome these limitations and establish safe and efficient dosage guidelines for more extensive therapeutic uses.

Only 14 countries have research published on ethnobotanical uses of *P. thonningii*, out of 46 countries in Sub-Saharan Africa. The unequal distribution indicates that there is a lack of thorough research on the ethnobotanical uses *P. thonningii* in most Sub-Saharan African nations. In contrast, nations like Nigeria have a more established interest in this plant, possibly reflecting a combination of cultural practices, biodiversity, and existing research infrastructure that supports ethnopharmacological studies (Ndodza et al., 2020; Danjuma et al. (2022); Lawal et al., 2022; Sidiq et al., 2022; Kankara et al., 2022; Evbuomwan et al., 2023; Falana et al., 2023). Therefore, to create representative and comprehensive ethnobotanical knowledge, more significant research initiatives must be undertaken to understand and record traditional uses of *P. thonningii* throughout the continent. The progress of the 14 countries in the number of publications on *P. thonningii* shows a committed effort to determine the advantages of *P. thonningii* within the healthcare context.

The studies used participants from local communities to conduct ethnobotanical and ethnopharmacological surveys. The largest group of participants was made up of traditional medicinal practitioners/healers accounting for 54% of participants across studies, followed by herbalists (14%) (Chegaing et al., 2020; Jeanne et al., 2020; Kola et al., 2020; Dossou et al., 2021; Akwongo et al., 2022; Toafode et al., 2022; Traore et al., 2022; Kankara et al., 2022; Kroa et al., 2022; Valentin et al., 2022; Sidiq et al., 2022; Bamogo et al., 2023; Evbuomwan et al., 2023; Herve et al., 2023; Masumbu et al., 2023; Valentin et al., 2023; Valentin et al., 2024). The results revealed that the latter participants play an important role in documenting knowledge of *P. thonningii,* and this underscores their vital role in transmitting and preserving ethnomedicinal knowledge. Often passed down from generation to generation, this knowledge remains a critical resource in places where conventional healthcare may be inaccessible (Cyril et al., 2021). The involvement of these primary knowledge holders allowed the studies to access detailed information on plant parts used, specific applications, preparation methods, and in-depth knowledge of therapeutic practices (Karaköse, 2022; Şen et al., 2022; Güler et al., 2021; Mahomoodally, 2013).

The parts of the plant commonly used were the leaves, bark, roots, and stem bark. In traditional African medicine, different plant parts are believed to have different therapeutic potentials. Cultural beliefs, plant sustainability, and recent scientific findings majorly influence this. For example, bark and leaves are commonly chosen because they are easily accessible and sustainable harvesting. After all, removing roots can be harmful to plant existence.

Furthermore, plant parts used to make remedies determine the preparation methods to be used such as decoction, maceration, powder, and juice. The most reported method was decoction, followed by maceration and powder (Chegaing et al., 2020; Kola et al., 2020; Valentin et al., 2020; Dossou et al., 2021; Dery et al., 2023; Akwongo et al., 2022; Bolou et al., 2022; Kroa et al., 2022; Traore et al., 2022; Valentin et al., 2023; Masumbu et al., 2023). A decoction is the boiling of plant material to extract water-soluble compounds, and this approach is common in traditional medicine to concentrate active ingredients (Akwongo et al., 2022). While maceration and powdering do not require heat, this may be because it is preserving heat-sensitive compounds. These methods reflect cultural preferences, and they allow for various applications such as oral ingestion, topical use, or inhalation (Kola et al., 2020; Akwongo et al., 2022). The latter diversity in administration indicates the versatility of this plant in treating a wide range of conditions/ailments from infectious diseases to chronic problems.

Commonly treated ailments include malaria, liver diseases, wounds, snakebites, gastrointestinal issues, and respiratory ailments (Chegaing et al., 2020; Ouédraogo et al., 2020; Valentin et al., 2020; Toafode et al., 2021; Dery et al., 2023; Evbuomwan et al., 2023). The ailments above are common in sub-Saharan Africa; hence, the traditional healers, etc., found a remedy using *P. thonningii* to cure/treat these diseases. For instance, there is a high prevalence of malaria in Nigeria (WHO, 2023), which is consistent with the fact that Nigeria was one of the leading countries in conducting research on this plant. The decoction of *P. thonningii* has been demonstrated to have antimalarial, antimicrobial, anti-inflammatory, cardioprotective, and hepatoprotective effects and it is known for its nephroprotective, anthelmintic, and antileishmanial activities (D’Almeida et al., 2024; Karaköse, 2022; Şen et al., 2022). While macerated or crushed are used to externally treat wounds, ulcers, and skin disorders (Toafode et al., 2022; Dery et al., 2023; Masumbu et al., 2023; Herve et al., 2023; Valentin et al., 2023). This treatment method is the same as the Western wound care protocol, which involves directly applying natural substances to afflicted areas. Furthermore, *P. thonningii* was used to treat cancer, diabetes, and high blood pressure, inflammatory and immune-modulating disorders, among other chronic conditions (Masumbu et al., 2023; Valentin et al., 2023).

The studies have revealed the potential of *P. thonningii* as a source of novel bioactive compounds that could assist in drug discovery, especially now because most microorganisms have developed resistance against most drugs. Literature has revealed phytochemical constitutes found in *P. thonningii* that have medicinal properties such as saponins, tannin, flavonoids, phenols, steroids, alkaloids, terpenoids, anthraquinone, reducing sugar, cardiac glycosides, resins, balsams, and volatile oil (Kwaji et al., 2010; Madara et al., 2010; Egharevba and Kunle, 2010; Ukwuani et al., 2012; Ighodaro et al., 2012). In addition, some novel compounds such as anthocyanidin-3-glycosides (Hassan et al., 2019), 6-hydroxylated flavonols (Antia et al., 2006), and flavonol-3-O-glucoside-7-O-rhamnoside) (Muhammad et al., 2015) were isolated from the 60% methanol leaf extract and showed to have antimicrobial activity (Akinpelu and Kolawole, 2004).

Despite the medical health benefits, studies have revealed that excessive consumption of this plant can cause adverse effects. (Uwagie-Ero et al., 2018). indicated that methanolic and aqueous stem bark extract administered 200 mg/kg body weight caused high serum levels of the liver enzyme (alanine aminotransferases and aspartate). Another study reported that aqueous extract of the plant induced cytoarchitectural changes in the kidneys of rats. Rats administered with 1 mg/kg body weight of the latter extract following orally consumption of 28 days showed vacuoles observed glomeruli with increased/bigger glomerular spaces (Cyril et al., 2021).

## Conclusion

The therapeutic uses of *P. thonningii* reveal a deep knowledge of symptom relief and disease management within Sub-Saharan African traditional medicinal practice. Many of the ailments, such as malaria, gastrointestinal infections, and skin diseases treated by *P. thonningii* were associated with their high prevalence in Sub-Saharan Africa, especially in Nigeria. The plant part commonly used was leaves and most remedies were prepared as decoction. This shows that traditional healers have, over centuries, refined the efficacy of *P. thonningii* for treating regionally prevalent ailments. This review emphasizes how crucial it is to preserve indigenous knowledge and investigate how it could influence contemporary medical procedures, particularly in places with few healthcare resources. Despite its potential, the lack of a standardized procedure to prepare remedies and dosages is concerning because it may lead to toxicity. Therefore, there is a need for interdisciplinary research merging ethnobotanical knowledge, pharmacological assessments, toxicological assays, and clinical trials. The latter studies will assist policymakers in developing evidence-based policies that are essential for understanding its uses across diverse cultures and settings. In addition, addressing the limitations of English-language sources will provide a more comprehensive understanding.

## Data Availability

The original contributions presented in the study are included in the article/Supplementary Material, further inquiries can be directed to the corresponding author.

## References

[B1] Abubakar I. B. Malami I. Muhammad A. Salihu Shinkafi T. Shehu D. Maduabuchi Aja P. (2024). A review of the medicinal uses and biological activity of Piliostigma thonningii (Schum). Milne-Redh. RPS Pharm. Pharmacol. Rep. 3 (1), rqae004. 10.1093/rpsppr/rqae004

[B2] Afolayan M. Srivedavyasasri R. Asekun O. T. Familoni O. B. Orishadipe A. Zulfiqar F. (2018). Phytochemical study of Piliostigma thonningii, a medicinal plant grown in Nigeria. Med. Chem. Res. 27, 2325–2330. 10.1007/s00044-018-2238-1 30319238 PMC6181138

[B3] Akinpelu D. A. Kolawole D. O. (2004). Phytochemical and antimicrobial activity of leaf extract of Piliostigma thonningii, Schum. 64–70.

[B4] Akwongo B. Katuura E. Nsubuga A. M. Tugume P. Andama M. Anywar G. (2022). Ethnobotanical study of medicinal plants utilized in the management of candidiasis in Northern Uganda. Trop. Med. Health 50 (1), 78. 10.1186/s41182-022-00471-y 36242066 PMC9569084

[B5] Antia B. S. Akpan E. J. Okon P. A. Umoren I. U. (2006). Nutritive and anti-nutritive evaluation of sweet potatoes (Ipomoea batatas) leaves. Pak. J. Nutr. 5, 166–168. 10.3923/pjn.2006.166.168

[B6] Arksey H. O’malley L. (2005). Scoping studies: towards a methodological framework. Int. J. Soc. Res. Methodol. 8 (1), 19–32. 10.1080/1364557032000119616

[B7] Bamogo R. Nikièma A. S. Belem M. Thiam M. Diatta Y. Dabiré R. K. (2023). Cross-sectional ethnobotanical survey of plants used by traditional health practitioners for snakebite case management in two regions of Burkina Faso. Phytomedicine Plus 3 (3), 1–9. 10.1016/j.phyplu.2023.100471

[B57] Bashige C. V. Bakari A. S. Okusa N. P. Kahumba B. J. Duez P. Lumba S. J. B. (2020). Self-medication with antimalarials drugs in Lubumbashi city (DR Congo). GSC Biol. Pharm. Sci. 12, 7–20.

[B58] Bashige V. C. Pierre K. I. Manya M. M. Mushagalusa F. (2022). Ethnobotanical study of plants used by traditional healers in Lubumbashi (Democratic Republic of Congo) in the management of typhoid fever. GSC Biol. Pharm. Sci. 21 (1), 265–286. 10.30574/gscbps.2022.21.1.0403

[B59] Bashige C. V. Philippe O. N. Melman M. Henry M. M. Salvius B. A. Baptiste L. S. J. (2024). Ethnomedical knowledge of plants used in alternative medicine to treat hemorrhoidal diseases in Lubumbashi, Haut-Katanga province, Southern Democratic Republic of Congo. BMC Complement. Med. Ther. 24 (1), 1–33. 39394139 10.1186/s12906-024-04646-4 PMC11468376

[B9] Bingham M. G. Willemen A. Wightman N. Wursten B. T. Ballings P. Hyde M. A. (2025). Flora of Zambia: species information: Piliostig. thonningii. Available online at: https://www.zambiaflora.com/speciesdata/imagedisplay.php?species_id=126830&image_id=1 (Accessed February 18, 2025).

[B10] Bolou G. E. Tra B. B. Yao K. Bouagnon J. J. Lidji C. S. N’guessan C. D. (2022). Inventory of plants used in the treatment of viral diseases, sold on markets in the district of Abidjan. GSC Biol. Pharm. Sci. 19 (1), 78–90. 10.30574/gscbps.2022.19.1.0132

[B11] Chegaing S. P. Mefokou D. Y. Tangue B. T. Sokoudjou J. B. Menoudji S. T. Kamsu G. T. (2020). Contribution to the ethnobotanical inventory of medicinal plants used for the treatment of typhoid fever in Adamaoua region, Cameroon. Int. J. Biol. Chem. Sci. 14 (9), 3078–3096. 10.4314/ijbcs.v14i9.9

[B12] Chiribagula V. B. Ndjolo Philippe O. Mboni H. M. Mushagalusa Kasali F. (2023). Ethnomedicinal knowledge of plants used in nonconventional medicine in the management of diabetes mellitus in kinshasa (democratic republic of the Congo). Evid. Based Complement. Alternat. Med. 2023 (1), 4621883. 10.1155/2023/4621883 37771953 PMC10533323

[B13] Codo T. N. M. Oppong Bekoe E. Vissiennon Z. Ahyi V. Vissiennon C. Fester K. (2022). Ethnomedicinal information on plants used for the treatment of bone fractures, wounds, and sprains in the northern region of the republic of Benin. Evid. Based Complement. Alternat. Med. 2022 (1), 8619330. 10.1155/2022/8619330 36588593 PMC9797300

[B14] Cyril O. Jonathan E. C. Chiedu O. F. (2021). Piliostigma thonningii (Fabaceae): a Comprehensive review on its traditional medicinal uses, phytochemistry, pharmacology and toxicology. Sch. Int. J. Biochem. 4, 66–81. 10.36348/sijb.2021.v04i07.001

[B15] D’Almeida S. A. Gbomor S. E. Osaio-Kamara B. Olagunju M. T. Abodunrin O. R. Foláyan M. O. (2024). A scoping review of the use of traditional medicine for the management of ailments in West Africa. PloS one 19 (7), e0306594. 10.1371/journal.pone.0306594 38976677 PMC11230574

[B16] Danjuma J. Abubakar I. B. Nwaogu J. Muhamamd A. Malami I. Abdulhamid A. (2022). Ethnomedicinal study and *in vitro* validation of medicinal plants used for treating Jaundice in Zuru emirate of Kebbi State, Nigeria. Ann. Sci. Technol. 7 (2), 29–40. 10.2478/ast-2022-0007

[B17] Dery G. Dzitse S. Tom-Dery D. (2023). Ethnobotanical survey of medicinal plants in Sissala East municipality of the upper West region, Ghana. Phytomedicine Plus 3 (3), 100461. 10.1016/j.phyplu.2023.100461

[B18] Diop A. L. Malan D. F. Kougbo M. D. (2022). Perception of malaria and cultural diversity of antimalarial plants in three sympatric communities: agni, Akyé and Ga in the District of Alépé, Côte d’Ivoire. Asian J. Ethnobiol. 5 (1). 10.13057/asianjethnobiol/y050101

[B19] Dossou A. J. Fandohan A. B. Djossa A. B. Assogbadjo A. E. (2021). Diversity and knowledge of plants used in the treatment of snake bite envenomation in Benin. Ethnobot. Res. Appl. 21, 1–20. 10.32859/era.21.48.1-20

[B20] Dossou A. J. Fandohan A. B. Omara T. Gbenou J. (2022). Traditional knowledge and phytochemical screening of plants used in snakebite prevention in Benin. Bull. Natl. Res. Centre 46 (1), 160. 10.1186/s42269-022-00851-8

[B21] Egharevba H. O. Kunle F. O. (2010). Preliminary phytochemical and proximate analysis of the leaves of Piliostigma thonningii (Schumach.) Milne-Redhead. Ethnobot. Leafl. 2010 (5), 2.

[B22] Evbuomwan I. O. Stephen Adeyemi O. Oluba O. M. (2023). Indigenous medicinal plants used in folk medicine for malaria treatment in Kwara State, Nigeria: an ethnobotanical study. BMC Complementary Med. Ther. 23 (1), 324. 10.1186/s12906-023-04131-4 PMC10504731 37716985

[B23] Falana M. B. Nurudeen Q. O. Salimon S. S. Abubakar I. B. (2023). Ethnopharmacological survey of medicinal plants used in the management of skin-related conditions in ilorin, north-central, Nigeria. Traditional Integr. Med. 10.18502/tim.v8i1.12404

[B24] Güler O. Polat R. Karaköse M. Çakılcıoğlu U. Akbulut S. (2021). An ethnoveterinary study on plants used for the treatment of livestock diseases in the province of Giresun (Turkey). South Afr. J. Bot. 142, 53–62. 10.1016/j.sajb.2021.06.003

[B25] Hailemariam M. B. Woldu Z. Asfaw Z. Lulekal E. (2021). Ethnobotany of an indigenous tree piliostigma thonningii (Schumach.) milne-redh. (fabaceae) in the arid and semi-arid areas of South omo zone, southern Ethiopia. J. Ethnobiol. Ethnomedicine 17, 44–48. 10.1186/s13002-021-00469-6 PMC8285841 34273997

[B26] Hassan L. G. Onwuasoanya A. H. Onwuasoanya S. C. Kolawole S. I. Ogbiko C. (2019). Nutritional composition, physicochemical and functional propertiesofpeeledandunpeeleddennettia tripetala (pepperfruit). GSJ. 7 (4).

[B27] Herve B. Ngameni B. Tembe E. A. Anih M. G. Borgia N. N. Fokunang C. N. (2023). Ethnobotanical and ethnopharmacological survey of herbal products of pharmaceutical importance for chronic wound management in bankim district of adamaoua region of Cameroon. J. Adv. Med. Pharm. Sci. 25 (7), 43–57. 10.9734/jamps/2023/v25i7632

[B28] Ighodaro O. M. Agunbiade S. O. Omole J. O. Kuti O. A. (2012). Evaluation of the chemical, nutritional, antimicrobial and antioxidant-vitamin profiles of piliostigma thonningii leaves (Nigerian species). Res. J. Med. Plant 6 (7), 537–543. 10.3923/rjmp.2012.537.543

[B29] Jeanne K. A. Dominique T. K. Alerte K. O. M. Kaddy R. N. A. Kroa E. Trésor D. M. (2020). Ethnopharmacological study of plants used against malaria by traditional healers in the department of bouna, north-eastern Côte d'Ivoire. J. Adv. Med. Pharm. Sci., 22(10), 11–22. 10.9734/jamps/2020/v22i1030196

[B30] Kahumba Byanga J. Jb D. P. Philippe O. N. J K. B. Pierre D. Jb L. S. (2020). Ethnobotanical study of plants used as antimalarial in traditional medicine in Bagira in Eastern RD Congo. J. Pharmacogn. Phytochemistry 9 (4), 01–14. 10.22271/phyto.2020.v9.i4a.11661

[B31] Kankara S. S. Nuhu A. I. Bindawa K. A. Haruna M. R. Bello A. Abubakar I. B. (2022). Indigenous traditional knowledge of medicinal plants used for the management of HIV/AIDS opportunistic infections in Katsina State, Nigeria. Ethnobot. Res. Appl. 23, 1–7. 10.32859/era.23.35.1-17

[B32] Karaköse M. (2022). An ethnobotanical study of medicinal plants in Güce district, north-eastern Turkey. Plant Divers. 44 (6), 577–597. 10.1016/j.pld.2022.03.005 36540712 PMC9751085

[B33] Kola P. Metowogo K. Kantati Y. T. Lawson-Evi P. Kpemissi M. El-Hallouty S. M. (2020). Ethnopharmacological survey on medicinal plants used by traditional healers in central and kara regions of Togo for antitumor and chronic wound healing effects. Evid. Based Complement. Alternat. Med. 2020 (1), 6940132. 10.1155/2020/6940132

[B34] Kroa E. Soumahoro A. Kouamé B. Y. Tiembre I. Yobouet M. K. (2022). Antimalarial and antianemic medicinal plants used by traditional medicine practitioners and the populations of the Korhogo 1 health district (Poro Region, Ivory Coast). GSC Biol. Pharm. Sci. 19, 154–171. 10.30574/gscbps.2022.19.1.0129

[B35] Kwaji A. Bassi P. U. Aoill M. Nneji C. M. Ademowo G. (2010). Preliminary studies on Piliostigma thonningii Schum leaf extract: phytochemical screening and *in vitro* antimalarial activity. Afr. J. Microbiol. Res. 4 (9), 735–739.

[B36] Lawal I. O. Rafiu B. O. Ale J. E. Majebi O. E. Aremu A. O. (2022). Ethnobotanical survey of local flora used for medicinal purposes among indigenous people in five areas in Lagos State, Nigeria. Plants 11 (5), 633. 10.3390/plants11050633 35270103 PMC8912796

[B37] Madara A. A. Ajayi J. A. Salawu O. A. Tijani A. Y. (2010). Anti-malarial activity of ethanolic leaf extract of Piliostigma thonningii Schum.(Caesalpiniacea) in mice infected with Plasmodium berghei berghei. Afr. J. Biotechnol. 9 (23), 3475–3480.

[B38] Mahomoodally M. F. (2013). Traditional medicines in Africa: an appraisal of ten potent African medicinal plants. Evid. Based Complement. Alternat. Med. 2013 (1), 617459. 10.1155/2013/617459 24367388 PMC3866779

[B39] Masumbu F. F. Mwamatope B. Tembo D. Mwakikunga A. Kamanula J. (2023). Ethnobotanical survey of medicinal plants claimed by traditional herbal practitioners to manage cancers in Malawi. J. Herb. Med. 42, 100796. 10.1016/j.hermed.2023.100796

[B40] Mogale M. M. Raimondo D. C. VanWyk B. E. (2019). The ethnobotany of Central Sekhukhuneland, South Africa. South Afr. J. Bot. 122, 90–119. 10.1016/j.sajb.2019.01.001

[B41] Mohlakoana M. Moteetee A. (2021). Southern african soap plants and screening of selected phytochemicals and quantitative analysis of saponin content. Resources 10 (10), 96. 10.3390/resources10100096

[B42] Moraa O. F. (2023). “ *In vivo* anti-obesity effects and phytochemical profiles of dichloromethane stem bark extracts of *piliostigma thonningii (schum)* and *lonchocarpus eriocalyx (harms)* in mice,” in Doctoral dissertation, school of pure and applied sciences (Kenya: kenyatta university).

[B43] Muhammad S. Umar K. J. Sani N. A. Muhammad S. (2015). Evaluation of nutritional and antinutritional profile of ginger bread plum (Neocarya macrophtlla) seed kernel. Int. J. Sci. Technol. 4, 361–367.

[B44] Namukobe J. Lutaaya A. Asiimwe S. Byamukama R. (2021). Ethnobotanical survey of medicinal plant species used by communities around Mabira and Mpanga Central Forest Reserves, Uganda. Trop. Med. Health 49 (1), 1–10. 10.1186/s41182-021-00341-z 34187581 PMC8243914

[B45] Nodza G. I. Onuminya T. O. Ogbu P. E. Agboola O. O. Ogundipe O. T. (2020). Ethnobotanical survey of medicinal plants used in treating snakebites in Benue. Nigeria. Ann. West Univ. Timiş. Ser. Biol. 23 (2), 147–158.

[B46] Orwa C. Mutua A. R. Kindt R. J. (2009). Piliostigma thonningii (schum) milne-redh. Aggroforestry Database 4, 1–5.

[B47] Ouédraogo L. Endl J. Sombié P. A. Schaefer H. Kiendrebeogo M. (2020). Ethnobotanical use and conservation assessment of medicinal plants sold in markets of Burkina Faso. Ethnobot. Res. Appl. 20, 1–25. 10.32859/era.20.39.1-25

[B48] Şen G. Akbulut S. Karaköse M. (2022). Ethnopharmacological study of medicinal plants in Kastamonu province (Türkiye). Open Chem. 20 (1), 873–911. 10.1515/chem-2022-0204

[B49] Sidiq L. O. Segun P. A. Ogbole O. O. (2022). Medicinal plants used in four local government areas of south-western Nigeria for the management of diabetes and its comorbidities: an ethnopharmacological survey. J. Phytomedicine Ther. 21 (1), 728–746. 10.4314/jopat.v21i1.1

[B50] Tittikpina N. K. Ejike C. E. Estevam E. C. Nasim M. J. Griffin S. Chaimbault P. (2016). Togo to go: products and compounds derived from local plants for the treatment of diseases endemic in sub-saharan Africa. Afr. J. traditional, complementary Altern. Med. 13 (1), 85–94. 10.21010/ajtcam.v13i1.12

[B51] Traore M. S. Camara A. Balde M. A. Diallo M. S. Barry N. S. Balde E. S. (2022). Ethnobotanical survey of medicinal plants used to manage hypertension in the Republic of Guinea. J. Pharm. and Pharmacogn. Res. 10 (5), 938–951. 10.56499/jppres22.1470_10.5.938

[B52] Tricco A. C. Lillie E. Zarin W. O'Brien K. K. Colquhoun H. Levac D. (2018). PRISMA extension for scoping reviews (PRISMA-ScR): checklist and explanation. Ann. Intern. Med. 169 (7), 467–473. 10.7326/M18-0850 30178033

[B53] Ukwuani A. Ihebunna O. Samuel R. M. Peni I. J. (2012). Acute oral toxicity and antiulcer activity of Piliostigma thonningii leaf fraction in albino rats. Bull. Env. Pharmacol. Life Sci. 2, 41–45.

[B54] Uwagie-Ero E. A. Nwaehujor C. O. Abiaezute C. N. Ocheja O. B. Ekeolu O. K. Asuzu I. U. (2018). D-3-O-methylchiroinositol (from Pilostigma thonningii) ameliorates cadmium chloride (CdCl2)-induced toxicity in male reproduction. J. Appl. Sci. Environ. Manag. 22 (6), 959–965. 10.4314/jasem.v22i6.20

[B56] World Health Organization (2023). Technical consultation to review the effectiveness of rectal artesunate used as pre-referral treatment of severe malaria in children: meeting report, 18–19 October 2022. World Health Organization.

