# Supplementary material for: Scoping review of ethnobotanical studies on Piliostigma thonningii (Schumach.) Milne-Redh. in Sub-Saharan Africa
Source: Front Pharmacol. 2025 Apr 16;16:1575548. doi: 10.3389/fphar.2025.1575548 (PMC12041768; doi:10.3389/fphar.2025.1575548)
Supplement: Supplementary file 1 [file Table1.docx]

| **Data charting**  Table 1: Ethnobotanical and ethnopharmacological studies of published manuscripts on *P. thonningii* in sub–Saharan Africa. | | | | | | |
| --- | --- | --- | --- | --- | --- | --- |
| S/NO | Author(s) | Year of publication | Title | Country | Study design | Study aim |
|  | Hailemariam, Woldu, Asfaw, & Lulekal | 2021 | Ethnobotany of an indigenous tree Piliostigma thonningii (Schumach.) Milne Redh. (Fabaceae) in the arid and semi-arid areas of South Omo Zone, southern Ethiopia | Southern Ethiopia | Ethnobotanical study | To assess and document the categoric ethnobotany of *Piliostigma thonningii* and the associated indigenous knowledge of the local people related to use, management practices, and the threatening factors in the South Omo Zone of southwestern Ethiopia |
|  | Codo Toafode, Oppong, Vissiennon, Ahyi, Vissiennon, & Fester | 2022 | Ethnomedicinal Information on Plants Used for the Treatment of Bone Fractures, Wounds, and Sprains in the Northern Region of the Republic of Benin | Northern Region of the Republic of Benin | Ethnobotanical study | To document the practices (diagnosis and materials) and traditional knowledge accumulated by healers in this region on their area of specialty |
|  | Akwongo, Katuura, Nsubuga, Tugume, Andama, Anywar, ... & Kakudidi | 2022 | Ethnobotanical study of medicinal plants utilized in the management of candidiasis in Northern Uganda | Uganda | Ethnobotanical study | This study explored ethnomedicinal plants as treatment option for candidiasis in Pader, Northern Uganda. |
|  | Chegaing, Mefokou, Tangue, Sokoudjou, Menoudji, Kamsu, & Gatsing | 2020 | Contribution to the ethnobotanical inventory of medicinal plants used for the treatment of typhoid fever in Adamaoua region, Cameroon | Cameroon | Ethnobotanical study | The present ethnobotanical survey was conducted to contribute to the knowledge of medicinal plants used for the treatment of typhoid fever in three sub divisions of Vina division, Adamawa Cameroon |
|  | [Evbuomwan](https://link.springer.com/article/10.1186/s12906-023-04131-4#auth-Ikponmwosa_Owen-Evbuomwan-Aff1-Aff2-Aff3), [Adeyemi](https://link.springer.com/article/10.1186/s12906-023-04131-4#auth-Oluyomi-Stephen_Adeyemi-Aff1-Aff2-Aff4) & [Oluba](https://link.springer.com/article/10.1186/s12906-023-04131-4#auth-Olarewaju_Michael-Oluba-Aff1-Aff2) | 2023 | Indigenous medicinal plants used in folk medicine for malaria treatment in Kwara State, Nigeria: an ethnobotanical study | Nigeria | Ethnobotanical study | The study was aimed at documenting the medicinal plants used for malaria treatment in folk medicine in Kwara State, Nigeria |
|  | Ouédraogo, Endl, Sombié, Schaefer, & Kiendrebeogo | 2020 | Ethnobotanical use and conservation assessment of medicinal plants sold in markets of Burkina Faso | Burkina Faso | Ethnobotanical survey | Identifying the most relevant species and their conservation status |
|  | Masumbu, Mwamatope, Tembo, Mwakikunga, & Kamanula | 2023 | Ethnobotanical survey of medicinal plants claimed by traditional herbal practitioners to manage cancers in Malawi | Malawi | Ethnobotanical study | This study documented medicinal plants that are claimed by traditional herbal practitioners (THPs) to manage specific types of cancers in Mzimba and Nkhata Bay districts of the northern region of Malawi |
|  | Dery, Dzitse, and Tom-Dery | 2023 | Ethnobotanical survey of medicinal plants in Sissala East municipality of the upper West region, Ghana | Ghana | Ethnobotanical study | This study investigated the ethnobotanical knowledge of the indigenes of the Sissala East Municipal District in the Upper West Region of Ghana |
|  | Kola, Metowogo, Kantati, Lawson-Evi, Kpemissi, El-Hallouty … & Aklikokou | 2020 | Ethnopharmacological Survey on Medicinal Plants Used by Traditional Healers in Central and Kara Regions of Togo for Antitumor and Chronic Wound Healing Effects | Togo | Ethnobotanical study | To record information on antitumor plants in central and Kara regions of Togo |
|  | Dossou, Fandohan, Omara, & Gbenou | 2022 | Traditional knowledge and phytochemical screening of plants used in snakebite prevention in Benin | Benin | Ethnobotanical study | To document the knowledge of traditional medicine specialists on the plants used to prevent snakebites or repel snakes in Benin |
|  | Bashige, C.V., Bakari, A.S, Okusa N.P, Kahumba B.J, Duez P and Lumbu S.J.B | 2020 | Ethnobotanical study of plants used as antimalarial in traditional medicine in Bagira in Eastern RD Congo | Democratic Republic of Congo | Ethnobotanical study | This transversal descriptive study was carried out to collect plants and recipes used in Bagira to treat malaria |
|  | Bashige, Pierre, Henry, & Mushagalusa | 2022 | Ethnobotanical study of plants used by traditional healers in Lubumbashi (Democratic Republic of Congo) in the management of typhoid fever | Democratic Republic of Congo | Ethnobotanical study | To identify plants and recipes used by traditional medicine practitioners (TMPs) in Lubumbashi to manage typhoid fever |
|  | Bashige, Philippe, Henry, Salvius, Suzanne, Kasali, & Baptiste | 2024 | Ethnomedical Knowledge of Plants Used in Nonconventional Medicine for Wound Healing in Lubumbashi, Haut-Katanga Province, DR Congo | Democratic Republic of Congo | Ethnobotanical study | To identify the plants used in Kinshasa to treat diabetes mellitus. |
|  | Jeanne, Tano, Kangah, Rasmane Na Ahou, Kroa, Trésor and Yavo | 2020 | Ethnopharmacological Study of Plants Used against Malaria by Traditional Healers in the Department of Bouna, North-Eastern Côte d'Ivoire | Côte d'Ivoire | Ethnobotanical study | The study aims at the fundamental knowledge of the plants used to treat malaria by the traditional healers of Bouna |
|  | Bamogo, Nikièma, Belem, Thiam, Diatta, & Dabiré | 2023 | Cross-sectional ethnobotanical survey of plants used by traditional health practitioners for snakebite case management in two regions of Burkina Faso | Burkina Faso | Ethnobotanical study | To identify medicinal plants used by traditional healers in the Hauts-Bassins and Southwest regions of Burkina Faso to manage snakebite cases and repel snakes |
|  | Herve, Ngameni, Tembe, Estella and Anih, Mbong and Borgia, & Fokunang. | 2023 | Ethnobotanical and Ethnopharmacological Survey of Herbal Products of Pharmaceutical Importance for Chronic Wound Management in Bankim District of Adamaoua Region of Cameroon | Cameroon | Ethnobotanical study | To identify plants and recipes used by some traditional healers in Bankim, Adamaoua Region of Cameroon for the management of chronic wounds |
|  | Bashige, [Philippe](https://onlinelibrary.wiley.com/authored-by/Philippe/Okusa+Ndjolo), [Manya,](https://onlinelibrary.wiley.com/authored-by/Henry/Manya+Mboni)  [Bakari](https://onlinelibrary.wiley.com/authored-by/Salvius/Bakari+Amuri) , [Masengu,](https://onlinelibrary.wiley.com/authored-by/Suzanne/Masengu+Kabeya)  [Kasali](https://onlinelibrary.wiley.com/authored-by/Kasali/F%C3%A9licien+Mushagalusa), [and Baptiste](https://onlinelibrary.wiley.com/authored-by/Baptiste/Lumbu+Simbi+Jean) | 2024 | Ethnomedical Knowledge of Plants Used in Nonconventional Medicine for Wound Healing in Lubumbashi, Haut-Katanga Province, DR Congo | Democratic Republic of Congo | Ethnobotanical study | The present study was carried out to survey the plants used in traditional medicine in Lubumbashi to treat wounds and to define their ethnomedical characteristics |
|  | Namukobe, J., Lutaaya, A., Asiimwe, S., & Byamukama, R. | 2021 | An Ethnobotanical Study of Medicinal Plants used in the Management of Dermatological Disorders in Buyende and Kayunga Districts, Uganda | Uganda | Ethnobotanical study | This study was done to document medicinal plants used in the management of dermatological disorders. |
|  | Nodza, Onuminya, Ogbu, Agboola, and Ogundipe. | 2020 | Ethnobotanical survey of medicinal plants used in treating snakebites in Benue, Nigeria. | Nigeria | Ethnobotanical study | The study presents an ethnobotanical survey of the medicinal plants used for treating snakes bites by the people of southern Benue, mainly the Idoma people |
|  | Dossou, A. J., Fandohan, A. B., Djossa, A. B., & Assogbadjo, A. E | 2021 | Diversity and knowledge of plants used in the treatment of snake bite envenomation in Benin | Benin | Ethnobotanical study | To contribute to a better knowledge of medicinal plants used in the treatment of snakebite envenomation in Benin. |
|  | Sidiq, Segun, and Ogbole | 2020 | Medicinal Plants Used in Four Local Government Areas of Southwestern Nigeria for the Management of Diabetes and Its Comorbidities: An Ethnobotanical Survey | Nigeria | Ethnobotanical study | Aimed at accessing and documenting the medicinal plants used in four local government of southwestern Nigeria for the management of diabetes and its comorbidities. |
|  | Kankara, Nuhu, Rabi’uHaruna, Bindawa, Abubakar and Bello | 2022 | Indigenous traditional knowledge of medicinal plants used for the management of HIV / AIDS opportunistic infections in Katsina State, Nigeria | Nigeria | Ethnobotanical study | An ethnobotanical study was conducted to document medicinal plants used for the management of HIV / AIDS opportunistic infections in three Local Government Areas (one from each Senatorial Zone) of Katsina State, Nigeria. |
|  | Kroa, Soumahoro, Kouamé, Tiembre, & Yobouet | 2022 | Antimalarial and antianemic medicinal plants used by traditional medicine practitioners and the populations of the Korhogo 1 health district (Poro Region, Ivory Coast) | Ivory Coast | Ethnobotanical study | To identify a list of medicinal plants used by Traditional Medicine Practitioners and the populations to treat malaria and anemia in the health district of Korhogo |
|  | Diop, Malan, and Kougbo | 2022 | Perception of malaria and cultural diversity of antimalarial plants in three sympatric communities: Agni, Akyé and Gwa in the District of Alépé,Côte d’Ivoire | Côte d’Ivoire | Ethnobotanical study | To determine how the communities with different histories perceive and manage malaria, a disease with a high prevalence rate in the Sub-Saharan region |
|  | Danjuma, Abubakar, Nwaogu, Muhamamd, Malami, and Abdulhamid | 2022 | Ethnomedicinal study and in vitro validation of medicinal plants used for treating Jaundice in Zuru emirate of Kebbi State, Nigeria | Nigeria | Ethnobotanical/experimental study | To investigate and document the herbal medicine used for treating jaundice in Zuru emirate. |
|  | Traore, Camara, Balde, Diallo, Barry, Balde, and Balde | 2022 | Ethnobotanical survey of medicinal plants used to manage hypertension in the Republic of Guinea | Republic of Guinea | Ethnobotanical survey | To identify the plants used in the traditional management of hypertension in Guinea |
|  | Lawal, Rafiu, Ale, Majebi and Aremu | 2022 | Ethnobotanical Survey of Local Flora Used for Medicinal Purposes among Indigenous People in Five Areas in Lagos State, Nigeria | Nigeria | Ethnobotanical survey | This research investigated and documented the use of medicinal plants in the management of various health conditions/diseases among local populations in Lagos State |
|  | Bolou, Tra, Yao, Bouagnon, Lidji, N’guessan, ... & Djaman | 2022 | Inventory of plants used in the treatment of viral diseases, sold on markets in the district of Abidjan | Côte d'Ivoire | Ethnobotanical survey | To inventory the plant species sold and collect ethnopharmacological data related to these plants |
|  | Falana, Nurudeen, Salimon, and Abubakar | 2023 | Ethnopharmacological Survey of Medicinal Plants Used in the Management of Skin-Related Conditions in Ilorin, North-Central, Nigeria | Nigeria | Ethnobotanical survey | This study was aimed at surveying the traditional medicinal values of some commonly used plants for the management of skin conditions by herbal practitioners in Ilorin metropolis |
|  | Mohlakoana and Moteetee | 2021 | Southern African Soap Plants and Screening of Selected Phytochemicals and Quantitative Analysis of Saponin Content | South Africa | Ethnobotanical/Experimental study | to compile a comprehensive list of plants used traditionally as soap substitutes in southern Africa and to assess the chemical properties of selected species |

Source: Authors
